# Functionalized β-Cyclodextrins Catalyzed Environment-Friendly Cycloaddition of Carbon Dioxide and Epoxides

**DOI:** 10.3390/ma16010053

**Published:** 2022-12-21

**Authors:** Qin Wen, Xuexin Yuan, Qiqi Zhou, Hai-Jian Yang, Qingqing Jiang, Juncheng Hu, Cun-Yue Guo

**Affiliations:** 1 College of Chemistry and Materials Science, South-Central Minzu University, Wuhan 430074, China; 2 School of Chemical Sciences, University of Chinese Academy of Sciences, Beijing 100049, China

**Keywords:** carbon dioxide fixation, functionalized β-cyclodextrins, solvent- and metal-free catalysis, environment-friendliness, cyclic carbonate

## Abstract

Ammonium, imidazole, or pyridinium functionalized β-cyclodextrins (β-CDs) were used as efficient one-component bifunctional catalysts for the coupling reaction of carbon dioxide (CO_2_) and epoxide without the addition of solvent and metal. The influence of different catalysts and reaction parameters on the catalytic performance were examined in detail. Under optimal conditions, Im-CD1-I catalysts functionalized with imidazole groups were able to convert various epoxides into target products with high selectivity and good conversion rates. The one-component bifunctional catalysts can also be recovered easily by filtration and reused at least for five times with only slight decrease in catalytic performance. Finally, a possible process for hydroxyl group-assisted ring-opening of epoxide and functionalized group- induced activation of CO_2_ was presented.

## 1. Introduction

CO_2_ has been attracting much attention because of its unique properties, such as nontoxicity, low cost, bio-renewability and C1 building block for organic synthesis, and so on [1,2,3,4]. During past decades, a great deal of efforts has been devoted to investigating efficient procedures for CO_2_ fixation to produce valuable products. The formation of cyclic carbonates via cycloaddition of CO_2_ with epoxides is among the most potential ways, and the obtained cyclic carbonates are used widely as aprotic polar solvents, precursors for polymerization reactions, electrolytes for lithium-ion batteries, and fine chemical intermediates [5,6,7,8,9]. Mechanistically, the Lewis acid center (e.g., metal center or H) ligates with the O of the epoxide to activate the epoxide substrate, while the Lweis base (e.g., halogen anion) acts as a nucleophilic reagent to open the ring of the epoxide so that the next steps such as CO_2_ insertion and intramolecular cyclization occur [10,11,12]. Therefore, various catalytic systems such as metal complexes [13,14,15,16,17,18,19], metal oxides [20,21,22,23], metalloporphyrins [24,25,26,27,28,29,30], ionic liquids [31,32,33,34,35], functional polymers [36,37,38,39,40], organocatalysts [41,42,43,44,45,46,47,48], metal-organic frameworks (MOFs) [49,50,51,52,53,54,55,56,57,58,59], mesoporous materials [60,61,62,63,64,65] and biomass [66,67,68,69,70,71,72,73,74,75,76,77,78,79,80,81,82,83,84,85,86] have been developed to promote such reaction up to now.

Though significant improvements in catalyst species have been achieved, some drawbacks such as scarce active sites and low carrier still remain to be overcome for effective chemical conversion of CO_2_ via its coupling reaction with epoxides [87,88]. Moreover, in many cases harsh reaction conditions (e.g., high pressure and/or high temperature) and participation of co-solvent/additive are generally required. Furthermore, accompanying issues of inherent corrosion, toxicity and environmental concerns associated with the use of metallic cations remain challenging. Therefore, it is very demanding to put forward efficient environment-friendly catalysts for the coupling reaction of carbon dioxide and epoxide.

Regarding catalysts reported so far, biomass-based environment-friendly catalysts have drawn great attention such as betaine [68], lecithin [70], sugarcane bagasse [77], chitosan-based [66,67,71,73,76], cellulose-based [69,72,74,75,78], chitin-supported [83] compounds, Xylan [89] and β-CD [90,91]. β-CD, a well-known inexpensive, stable, and readily available natural hydrogen bond donor, is of abundant hydroxyl groups. In 2008, Han et al. firstly reported an efficient catalytic system of β-CD/KI for CO_2_ cycloaddition with epoxides [90]. This work broadened the application of β-CD in CO_2_ fixation and more β-CD-based catalytic systems are highly expected. Unfortunately, there has been no substantial advance in this field until 2017 when an efficient [DBUH][PFPhO]/β-CD system was developed in Hou’s group [91]. However, functionalized β-CDs as efficient catalytic systems compared to other biological materials are still rarely reported. Therefore, ammonium functionalized bis-β-CDs as effective catalysts were synthesized in our group to catalyze the coupling reaction of CO_2_and epoxides [84].

Although the catalytic results are positive and encouraging, the synthesis and yield of modified bis-β-CDs are difficult. Young and Ng et al. synthesized a series of functionalized β-CDs by displacing 6-tosyl-β-CD with alkylamines, pyridinium or alkylimidazoles via easy ways [92]. Inspired by their work, we herein designed and synthesized a series of functionalized β-CDs to be as one-component catalysts, in which hydroxyl groups were functionalized as Lewis acid to activate the epoxide and halide ions were functionalized as Lewis base to promote the ring-opening step. The catalytic performance of newly synthesized, functionalized β-CD for the coupling reaction of CO_2_ and epoxides were systematically investigated without adding co-catalyst and solvent. Moreover, these β-CD based catalysts can be reused conveniently, which is important for developing practical processes. Furthermore, a synergistic mechanism involved hydroxyl group and halide ion was discussed based on the literatures and experimental results. This ecologically safe, simple, inexpensive catalytic system has potential of CO_2_ conversion at the industrial level in the future.

## 2. Materials and Methods

### 2.1. Chemicals and Analytical Methods

All the chemicals were purchased from Acros and used as received except for epoxides which were purified by distillation from CaH_2_ before use. A Bruker Al-400 MHz instrument manufactured by Bruker Technologies Switzerland Ltd., Fällanden, Switzerland, was used for recording NMR spectra using TMS as an internal standard.

### 2.2. Synthesis of Functionalized β-CDs

**Scheme 1:**
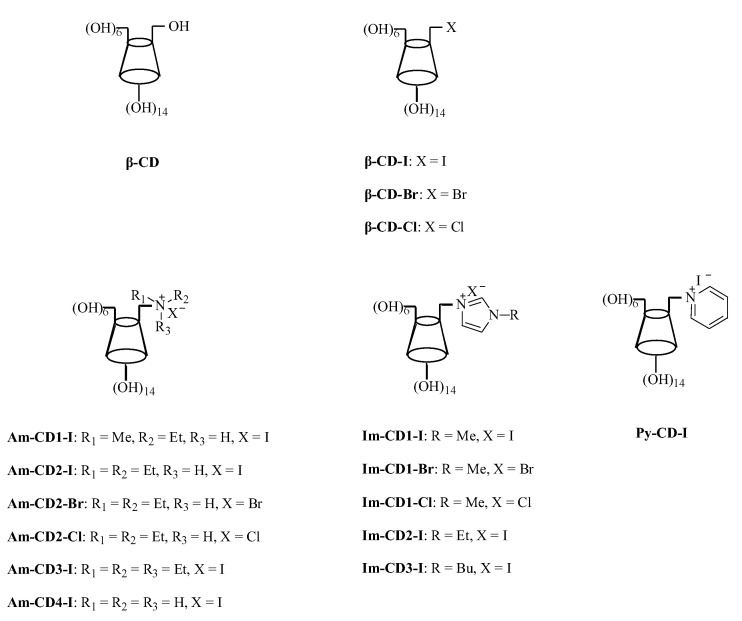
Structures of β-CD and various functionalized β-CDs.

The ammonium, imidazole, and pyridinium functionalized β-CDs (Scheme 1) were exactly synthesized and characterized according to previously reported methods [92]. After synthesis and purification following the reported procedures, these functionalizedβ-CDs were directly employed to initiate CO_2_ coupling reaction with epoxides.

### 2.3. General Procedure for Cyclic Carbonates Synthesis from Epoxides and CO_2_

Cycloaddition reaction of CO_2_ and epoxide was conducted in a 250 mL stainless steel autoclave. In a typical reaction, predetermined amounts of catalyst and epoxide were fed into the reactor, CO_2_ was then added into the reactor at certain pressure. The autoclave was sealed and then immersed into an oil bath at preset temperature with stirring. The reactor was cooled down in an ice-water bath after predesigned time and the unreacted CO_2_ was released slowly. The yield and selectivity are determined by ^1^H NMR characterization.

## 3. Results and Discussion

### 3.1. Effect of Reaction Parameters with Am-CD1-I

**Figure 1:**
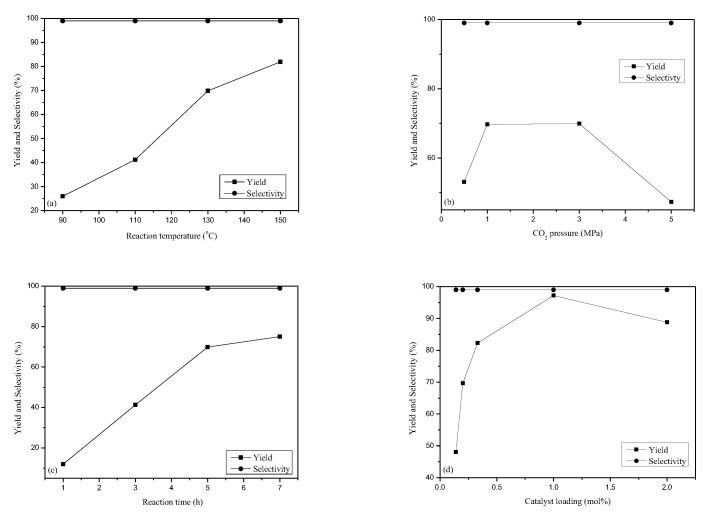
Effects of different reaction parameters on PC yield over Am-CD1-I: (**a**) Effects of reaction temperature, conditions: PO (5 mL, 71.5 mmol), Am-CD1-I (0.2 mol%), CO_2_ pressure 3 MPa, time 5 h; (**b**) Effects of CO_2_ pressure, conditions: PO (5 mL, 71.5 mmol), Am-CD1-I (0.2 mol%), reaction temperature 130 °C, time 5 h. (**c**) Effects of reaction time, conditions: PO (5 mL, 71.5 mmol), Am-CD1-I (0.2 mol%), reaction time 130 °C, CO_2_ pressure 3 MPa. (**d**) Effects of catalyst loading, conditions: PO (5 mL, 71.5 mmol), reaction time 130 °C, CO_2_ pressure 3 MPa, reaction time 5 h.

Reaction conditions were screened for optimizing the catalytic activity based on the ammonium functionalized β-CD Am-CD1-I and the coupling reaction of CO_2_ and propylene oxide (PO). The reaction conditions as collected in Figure 1 were standardized by observing the effect of reaction temperature, pressure, time, and catalyst loading on the yield of propylene carbonate (PC).

The reaction temperature was first investigated to test its effect on the PC yield. Figure 1a displays a strong effect of temperature on the PO conversion. A high reaction temperature is favorable for the synthesis of PC, indicating that the cycloaddition reaction was thermodynamically favorable [93,94]. To our satisfaction, low temperature appears insensitive to the PC selectivity in view of the fact that low temperature would be favorable for producing polycarbonate. Considering that polymerization of cyclic carbonates occurs at higher temperatures [95] and has adverse effect on the equipment, an optimal reaction temperature of 130 °C was selected for following studies.

Figure 1b reflects the influence of the CO_2_ pressure on the PC yield. Low pressures ranging from 0.5 to 1.0 MPa gave rise to increase in the PC yield. The PC yield decreased in high-pressure range (3.0–5.0 MPa) after a plateau from 1.0 to 3.0 MPa of CO_2_ pressure. Such phenomenon is observed as well in other catalytic systems [96,97,98]. It could be explained that in the low-pressure region, the increase in CO_2_ pressure enhanced PC yield due to higher CO_2_ concentration in the liquid phase. However, much higher CO_2_ pressure would lower the PC yield due to decreased PO concentration around the catalyst, this is not favorable for the cycloaddition because PO is another reactant [99,100]. As a result, a maximal PC yield was obtained.

The PC yield increased steadily with reaction time until 7 h and the coupling reaction proceeded rapidly within the first 5 h, and no appreciable increment in PC yield was observed thereafter (Figure 1c). This might originate from a hampered interaction between the catalyst and reactant due to the formation of PC [101]. A more viscous reaction system after prolonged reaction time was another negative factor disfavoring the activation of CO_2_. Thus, the reaction time of 5 h was chosen to be optimal. Increasing of the catalyst loading from 0.14 mol% to 1 mol% led to rising catalytic activity (Figure 1d). However, there was a decrease in the PC yield for the reaction conducted with 2 mol% catalyst, which may be from a hindered mass transfer due to excess catalyst. Thus, 1 mol% **Am-CD1-I** is optimum for this work and selected for subsequent research.

### 3.2. Effect of Reaction Parameters over Im-CD1-I

**Figure 2:**
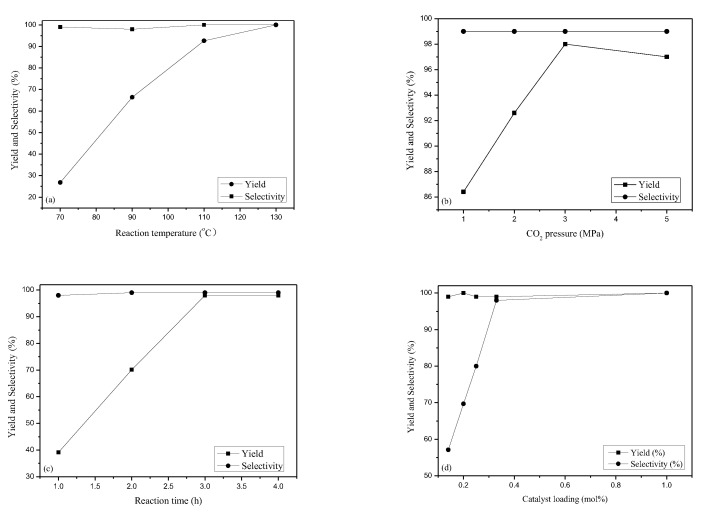
Effects of different reaction parameters on PC yield over Im-CD1-I: (**a**) Effects of reaction temperature, conditions: PO (5 mL, 71.5 mmol), Im-CD1-I (0.33 mol%), CO_2_ pressure 2 MPa, time 3 h; (**b**) Effects of CO_2_ pressure, conditions: PO (5 mL, 71.5 mmol), Im-CD1-I (0.33 mol%), reaction temperature 110 °C, time 3 h; (**c**) Effects of reaction time, conditions: PO (5 mL, 71.5 mmol), Im-CD1-I (0.33 mol%), reaction time 110 °C, CO_2_ pressure 3 MPa; (**d**) Effects of catalyst loading, conditions: PO (5 mL, 71.5 mmol),reaction time 110 °C, CO_2_ pressure 3 MPa, reaction time 3 h.

Inspired by the high performance of ammonium functionalized β-CD Am-CD1-I, the imidazole functionalized β-CD Im-CD1-I was also attempted to catalyze the cycloaddition of CO_2_ and PO. PC yields catalyzed by Im-CD1-I trended similarly to those in the case of using Am-CD1-I (Figure 2). The reaction temperature also affected the PC yield and 110 °C is chosen to be optimal (Figure 2a). The PC formation with Im-CD1-I correlated with CO_2_ pressure (Figure 2b). The peak PC yield appeared at 3 MPa and much higher CO_2_ pressure resulted in decreased yield. Moreover, prolonged reaction time exceeding 3 h failed in further increasing PC yield (Figure 2c). The PC yield at the low catalyst loading (0.14–0.33 mol%) rose with increasing catalyst loading, realizing a drastic elevation in PC yield. However, a further increase in catalyst loading contributed little to PC yield (Figure 2d). To sum up, the optimal condition of the cycloaddition over Im-CD1-I is 110 °C, 3 MPa, 3 h and 0.33 mol%.

Comparison of the optimal conditions for Am-CD1-I and Im-CD1-I (130 °C, 1 MPa, 5 h, 1 mol% Am-CD1-I vs. 110 °C, 3 MPa, 3 h, 0.33 mol% Im-CD1-I) found that ammonium functionalized β-CD Am-CD1-I required higher reaction temperature and more time, along with higher catalyst loading, while the imidazole functionalized β-CD Im-CD1-I only required relatively higher CO_2_ pressure, which may be due to the different solubility of the two functional β-CDs in the reaction system. In general, the optimal condition for Im-CD1-I is milder than that forAm-CD1-I, reflecting the superiority of imidazole functionalized β-CDs catalysts although Im-CD1-I and Am-CD1-I are both metal-, solvent- and cocatalyst-free.

### 3.3. Catalytic Performances of Various Catalysts

Under optimal conditions, solvent-free synthesis of PC from CO_2_ and PO catalyzed by ammonium, imidazole, and pyridinium functionalized β-CDs was investigated. As listed in Table 1, most of functionalized β-CDs afforded excellent selectivity for PC. The reason for such a high selectivity of this reaction is due to the tendency of the X^−^ ion to attack the C with small site resistance during the nucleophilic attack on the epoxide to open its ring. The details will be shown in the description of the mechanism section. For various catalysts, the catalytic activities correlated with their structures. The mono-6-halide-β-CDs can convert PO in quantitative yields (Table 1, entries 1–3), suggesting synergetic effect of rich hydroxyl groups and halide ions in the modified β-CDs. The synergetic effect of these two functional groups has been reported and testified using a DFT calculation by Zhang et al. [102]. Moreover, when β-CD was modified by ammonium, imidazole, and pyridinium, the catalyst performance was improved visibly. For the ammonium functionalized β-CDs (Table 1, entries 4–9), higher catalytic activities of Am-CD1-I and Am-CD2-I are attributed to bulky alkyl on the butyl amine group. The more-bulky butyl on the amine group might form a more flexible ion pair with I-, thus increasing its nucleophilicity and making it the most viable catalyst [103,104]. For both mono-6-halide-β-CDs and functionalized β-CDs, the activity for various halogen anions decreases in the order of I^−^ > Br^−^ > Cl^−^(Table 1, entries 1–3,5–7,13–15) probably owing to the leaving ability and nucleophilicity of the anion [105,106]. To study the effect of imidazole functionalized β-CD structure on the catalytic activity, a milder reaction condition was conducted afterwards. A longer alkyl chain endowed imidazole functionalized β-CD with higher catalytic performance (Table 1, entries 16–18) because a long alkyl chain may weaken electrostatic interaction, thus enhancing the nucleophilicity of anion [107]. The ammonium, imidazole, and pyridinium functionalizedβ-CDs played quite well in coupling reaction between CO_2_ and epoxides with much better performance compared with binary catalytic system β-CD/KI [90] or β-CD/TBAI [108], because our catalyst only required lower CO_2_ pressure and catalyst loading without adding metal and additive.

### 3.4. Recycling Test

**Figure 3:**
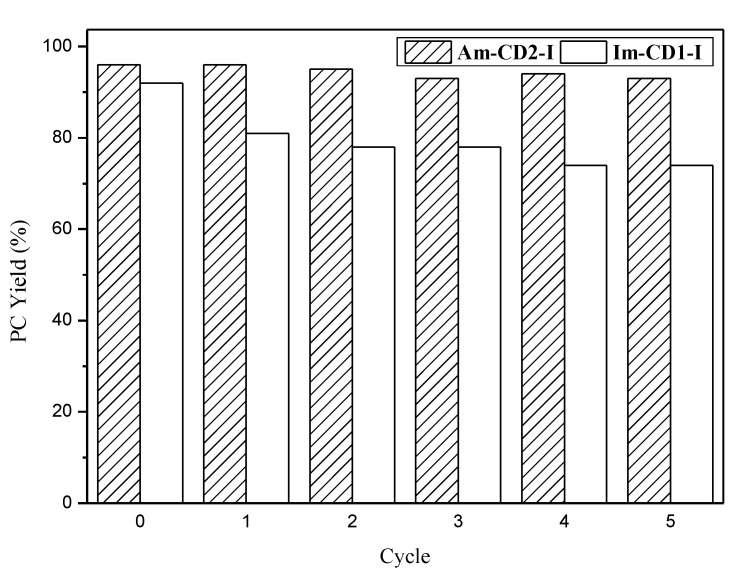
Catalyst recycling for coupling reaction catalyzed by Am-CD2-I and Im-CD1-I. Reaction condition for Am-CD2-I: PO (5 mL, 71.5 mmol), Am-CD2-I (1 mol%), CO_2_ pressure 1 MPa, reaction temperature 130 °C, time 5 h. Reaction condition for Im-CD1-I: PO (5 mL, 71.5 mmol), Im-CD1-I (0.33 mol%), CO_2_ pressure 3 MPa, reaction temperature 110 °C, time 3 h. The selectivity for target products are all > 99%.

**Figure 4:**
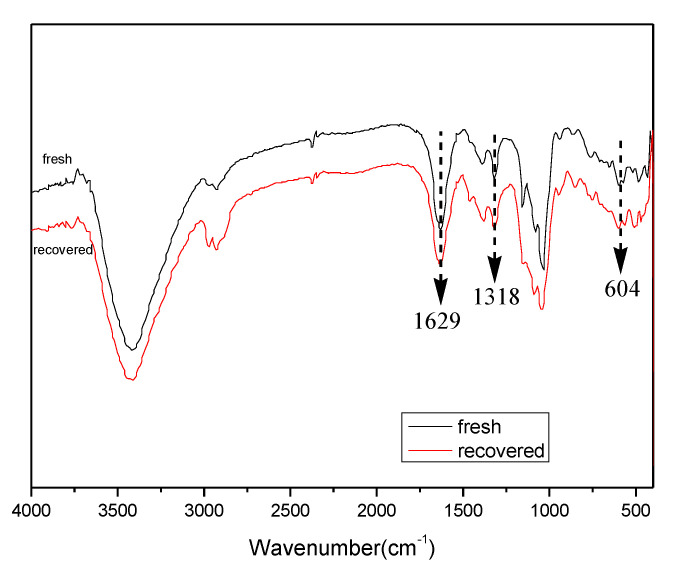
FT−IR spectra of Im-CD1-I and Im-CD1-I after five catalytic runs. Reaction condition: PO (5 mL, 71.5 mmol), Im-CD1-I (0.33 mol%), CO_2_ pressure 3 MPa, reaction temperature 110 °C, time 3 h.

A series of reaction recycles using Am-CD1-I and Im-CD1-I as catalysts were performed to investigate the stability of the catalyst for the cycloaddition reaction of PO with CO_2_ under each optimal condition. In each cycle, Am-CD1-I and Im-CD1-I were recovered via simple filtration, washed with acetone, dried in vacuo and directly reused for the next cycle. As Figure 3 presents, both Am-CD1-I and Im-CD1-I can be reused for at least 5 times without obvious loss in catalytic activity. In order to confirm the stability of Im-CD1-I (showing slight decrease after 5 times of recycling, Figure 3), the reused Im-CD1-I was characterized by FT-IR analysis. Strong characteristic bands assigned to the in-plane C-H deformation vibration and in-plane C-C and C-N stretching vibration of the imidazole ring (1629 and 1318 cm^−1^), along with the characteristic band of C-I bond at 604 cm^−1^ remain after reuse (Figure 4), indicating a very stable Im-CD1-I for this reaction. As shown in Figure 4, the structure of the catalyst was maintained after five times of reuse, which proved the stability and reusability of the synthesized catalyst, and the slight decrease in the catalytic effect after five times of use might be due to the partial loss of catalyst during the recycling process.

### 3.5. Cycloaddition of Various Epoxides and CO_2_

To probe the prospect and versatility of as-synthesized functionalized β-CD catalyst, the cycloaddition reaction of CO_2_ with various epoxides with Im-CD1-Iwas studied (Table 2). Im-CD1-I worked well towards various epoxides possessing both electron-withdrawing and electron-donating substituents, forming respective cyclic carbonates with excellent selectivity and good yields. For isobutyl oxide (Table 2, entry 7) and cyclohexene oxide (Table 2, entry 8), identical reaction conditions gave rise to relatively low yield possibly due to that a steric hindrance obstructed the nucleophilic attack of the epoxide while its coordination to the Lewis acid metal center benefited the yield [108,109,110,111]. The aliphatic substituted epoxides (including PO in Table 1) were transformed with CO_2_ to desired products in good yields. Especially, the activated epoxide epichlorohydrin was converted by as-designed catalysts and transformed into respective cyclic carbonate in good yield in 3 h (Table 2, entry 1). Surprisingly, aromatic substituted epoxide styrene oxide reacted with CO_2_ in a yield of 100% (Table 2, entry 3). Furthermore, the glycidyl ethers were turned into corresponding carbonates in good yields from 75 to 98% (Table 2, entries 4–6). It is also noteworthy that the Im-CD1-I catalyzed the diepoxides to produce respective bicyclic carbonates as well (Table 2, entries 10,11), raw materials for synthesizing non-isocyanate polyurethanes (NIPUs) without using toxic phosgene or isocyanates via the reaction with polyfunctional primary amines [112,113,114]. With increasing aliphatic chain length, the addition of CO_2_ was hindered because of chain folding or the fluidity of chains and the hindrance of methylene groups. Such phenomenon was also observed by Qin et al. [115,116,117].

### 3.6. Proposed Mechanism

**Scheme 2:**
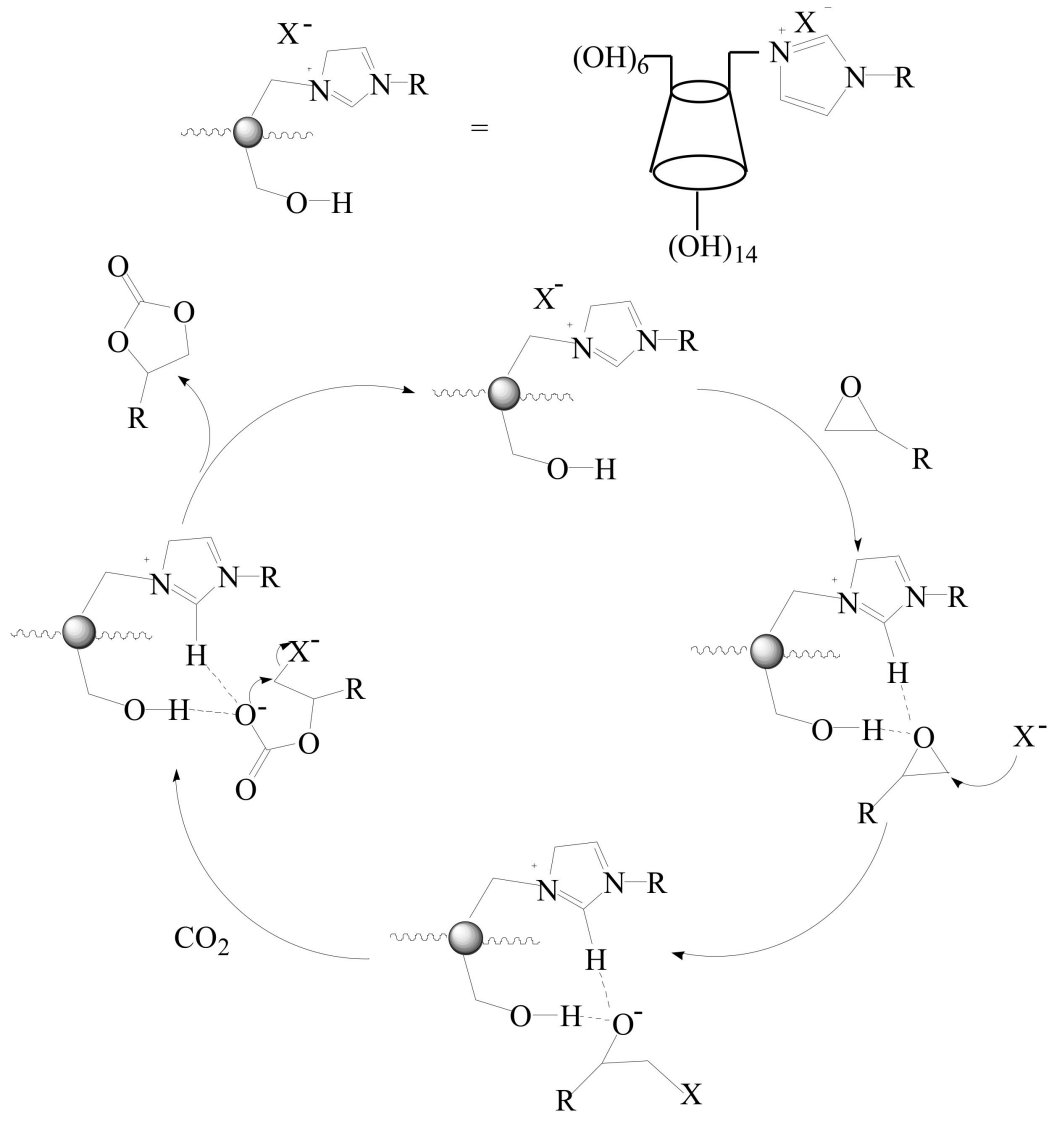
Proposed mechanism.

A mechanism is proposed for the functionalized β-CD catalyzed reaction as shown in Scheme 2 based on experimental results and literatures [118]. Firstly, the interaction between the epoxide oxygen and hydroxyl groups of Im-CD1-I promoted the polarization of the C-O bond in epoxide as reported in literature [119,120,121,122,123]. Simultaneously, CO_2_ was activated by functionalized group, such as imidazole herein. Moreover, the imidazolium cations could also stabilize the metal-alkoxide bond through charge interactions, which would help explain the superior performance of imidazole, ammonium, or pyridinium functionalized β-CDs than mono-6-halide-β-CDs. Subsequently, the nucleophilic halide anion attacked the less hindered carbon atom of epoxide followed by ring opening step to form an intermediate of oxygen anion. The oxygen anion intermediate then reacted with activated CO_2_ to form a carbonate anion, followed by an intramolecular ring-closure step to produce cyclic carbonate and regenerate the catalyst. According to this mechanism, the cooperative effect between the electrophile (hydrogen bond) and nucleophile (flexible halide anion) in the same catalyst molecules could effectively promote the coupling reaction in an eco-friendly mode without the introduction of metal, additive, and solvent [124,125].

### 3.7. Comparison of Different Biological Catalytic Systems

Catalytic activities of as-synthesized functionalized β-CDs in the coupling reaction of CO_2_ and PO are compared with those of other biological catalyst systems reported in literature [66,67,68,69,70,71,72,73,74,75,76,77,78,83,89,90,91]. Though the reaction conditions differ from each other to some extent (reaction temperature: 100–140 °C; CO_2_ pressure: 1.17–8 MPa; reaction time: 1–10 h; yield: 85–100%; TOF: 5–81 h^−1^), approximate comparison is reasonable. Compared with either β-CD-based catalytic systems [90,91], or other biological catalytic systems [66,67,68,69,70,71,72,73,74,75,76,77,78,83], the as-synthesized functionalized β-CDs Am-CD2-I, Im-CD1-I and Py-CD-I demonstrated better performance as indicated by significantly higher yield values (96% for Am-CD2-I and 98% for Im-CD1-I) than the yield of 85% for the Xylan/DBU catalytic system [89] in addition to metal- and cocatalyst-free conditions adopted in this work. The comprehensive catalytic performance of the present catalysts is also better than that of MOFs and metal oxides [22,49,54]. Overall, the functionalized β-CDs utilized in this work are among excellent catalysts in comparison to most of efficient biological catalyst systems reported so far.

## 4. Conclusions

A series of imidazole, ammonium, and pyridinium functionalized β-CDs were first employed as a one-component and recyclable catalyst for the coupling reaction between various epoxides and CO_2_ without the addition of metal, cocatalyst, and solvent. Excellent selectivity and high cyclic carbonate yields are realized under mild conditions. As disclosed by the mechanism, the reaction proceeded smoothly owing to a synergistic effect from abundant hydroxyl groups of β-CD and the halide anion of functional groups. These green, biocompatible, and non-toxic catalysts derived from inexpensive environment-friendly starting material β-CD have great potential in industrial application for the conversion of CO_2_.

## Figures and Tables

**Table 1:** Results of coupling reaction of CO_2_ and PO catalyzed by various catalysts ^a^.

Entry	Catalyst	Yield (%) ^b^	Selectivity (%) ^b^	TON ^c^	TOF (h^−1^) ^d^
1	β-CD-I	78	99	78	16
2	β-CD-Br	65	99	65	13
3	β-CD-Cl	60	99	60	12
4	Am-CD1-I	97	99	97	19
5	Am-CD2-I	96	99	96	19
6	Am-CD2-Br	89	99	89	18
7	Am-CD2-Cl	79	99	79	16
8	Am-CD3-I	90	99	90	18
9	Am-CD4-I	84	99	84	17
10 ^e^	β-CD-I	26	98	80	27
11 ^e^	β-CD-Br	20	99	61	20
12 ^e^	β-CD-Cl	3	99	9	3
13 ^e^	Im-CD1-I	98	99	297	99
14 ^e^	Im-CD1-Br	91	98	276	92
15 ^e^	Im-CD1-Cl	3	99	10	3
16 ^f^	Im-CD1-I	80	99	320	107
17 ^f^	Im-CD2-I	90	98	360	120
18 ^f^	Im-CD3-I	92	99	367	122
19 ^e^	Py-CD-I	89	99	271	90
20 ^g^	β-CD/KI	98	99	223	56

^a^ Reaction conditions: PO (5 mL, 71.5 mmol), catalyst 1 mol%, CO_2_ pressure 1 MPa, reaction temperature 130 °C, reaction time 5 h. ^b^ Determined by ^1^H NMR spectra analysis using TMS as an internal standard. ^c^ Turnover number for PC calculated as mole of PC produced per mole of catalyst. ^d^ Turnover frequency for PC calculated as mole of PC produced per mole of catalyst per hour. ^e^ Reaction conditions: PO (5 mL, 71.5 mmol), catalyst 0.33 mol%, CO_2_ pressure 3 MPa, reaction temperature 110 °C, reaction time 3 h. ^f^ Reaction conditions: Catalyst 0.25 mol%. ^g^ Reaction conditions: PO (22 mmol), β-CD 0.1 g, KI 2.5 mol%, CO_2_ pressure 6 MPa, reaction temperature 120 °C, reaction time 4 h.

**Table 2:** Cycloaddition between CO_2_ and various epoxides catalyzed by Im-CD1-I ^a^.

Entry	Epoxide	Product	Yield (%) ^b^	Selectivity (%) ^b^	TON ^c^	TOF (h^−1^) ^d^
1 ^e^	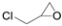	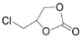	86	99	259	86
2	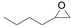	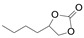	80	98	240	10
3	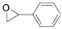	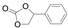	100	99	300	13
4	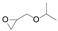	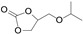	75	98	226	9
5	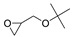	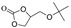	88	99	265	11
6	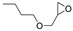	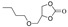	98	99	294	12
7	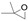	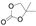	38	98	115	5
8	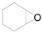	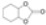	6	99	17	1
10	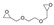	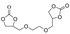	86	99	259	11
11	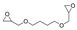	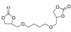	98	98	294	12

^a^ Reaction conditions: Im-CD1-I (0.33 mol%), epoxide (71.5 mmol), reaction temperature 110 °C, pressure 3 MPa, reaction time 24 h. ^b^ Determined by ^1^H NMR spectra analysis using TMS as an internal standard. ^c^ TON for cyclic carbonate calculated as mole of cyclic carbonate produced per mole of catalyst. ^d^ TOF for cyclic carbonate calculated as mole of cyclic carbonate produced per mole of catalyst per hour. ^e^ Reaction time 3 h.

## Data Availability

The data presented in this study are openly available in MDPI.
